# Homology-dependent recombination of large synthetic pathways into *E. coli* genome via λ-Red and CRISPR/Cas9 dependent selection methodology

**DOI:** 10.1186/s12934-020-01360-x

**Published:** 2020-05-24

**Authors:** Buli Su, Dandan Song, Honghui Zhu

**Affiliations:** State Key Laboratory of Applied Microbiology Southern China, Guangdong Provincial Key Laboratory of Microbial Culture Collection and Application, Guangdong Microbial Culture Collection Center (GDMCC), Guangdong Institute of Microbiology, Guangdong Academy of Sciences, Guangzhou, 510070 People’s Republic of China

**Keywords:** Metabolic engineering, Chromosomal integration, Lambda Red, CRISPR-Cas9, *Escherichia coli*

## Abstract

**Background:**

Metabolic engineering frequently needs genomic integration of many heterologous genes for biosynthetic pathway assembly. Despite great progresses in genome editing for the model microorganism *Escherichia coli*, the integration of large pathway into genome for stabilized chemical production is still challenging compared with small DNA integration.

**Results:**

We have developed a λ-Red assisted homology-dependent recombination for large synthetic pathway integration in *E. coli*. With this approach, we can integrate as large as 12 kb DNA module into the chromosome of *E. coli* W3110 in a single step. The efficiency of this method can reach 100%, thus markedly improve the integration efficiency and overcome the limitation of the integration size adopted the common method. Furthermore, the limiting step in the methylerythritol 4-phosphate (MEP) pathway and lycopene synthetic pathway were integrated into the W3110 genome using our system. Subsequently, the yields of the final strain were increased 106 and 4.4-fold compared to the initial strain and the reference strain, respectively.

**Conclusions:**

In addition to pre-existing method, our system presents an optional strategy for avoiding using plasmids and a valuable tool for large synthetic pathway assembly in *E. coli*.

## Background

*Escherichia coli* is a model microorganism usually used for synthetic biology and industrial applications [1]. Meanwhile, *E. coli* is known as one of the most promising host for the development of microbial cell factories [2]. Over the last decade, various metabolic engineering strategies, including overexpression of key genes, deletion of competitive pathways and chromosomal integrations, have been developed in *E. coli* to improve the metabolic flux and consequently increase production yields [3]. Novel approaches for introducing synthetic DNA modules, particularly large synthetic pathways, into *E. coli* would therefore greatly facilitate engineering processes.

The widely used methods for genome editing in *E. coli* were developed on the basis of λ-Red promoted homology-dependent recombination (HDR). Chromosomal integration of DNA modules with the size of about 2000 bp could be accomplished through λ-Red promoted HDR with high efficiency [4]. However, the recombination efficiency could decrease drastically for large DNA modules (> 2000 bp) and chromosomal integration of DNA module which was larger than 2500 bp using λ-Red promoted HDR was very difficult [5]. In particular, the elimination of antibiotic marker was inconvenient, and the residual FRT sites might bring about unexpected recombination in the genome of the edited strain. Various chromosomal integration strategies based on λ-Red system have been established for large DNA modules integration, including I-SceI cleavage-facilitated recombination [6], knock-in/knock-out (KIKO) vector mediated integration [7] and pSB1K3(FRTK) vector aided insertion [8]. However, the integration efficiency was significantly decreased when the size of the DNA modules was gradually increased. Currently, the maximum length of the integrated DNA modules was about 10 kb assisted by the λ-Red promoted HDR [5]. As an exceptional case, DNA modules (~ 15 kb) were divided into four segments (each ~ 3 kb) and then iteratively integrated into *E. coli* genome [9]. Furthermore, a 50 kb DNA module from *B. subtilis* 168, divided into seven segments (each ~ 6–7 kb), was inserted into *E. coli* genome through iterative integration [9]. This method could integrate large DNA fragment, which however required many rounds of integration to achieve large synthetic pathways integration.

In recent years, CRISPR/Cas9 has become the most widely used technology for genome editing in a variety of organisms [10]. In *E. coli*, the CRISPR-Cas9 technology was generally accomplished assisted by the λ-Red promoted HDR for chromosomal integration [11]. For example, DNA modules as large as 7 kb could be integrated in *E. coli* chromosome with > 60% efficiency using CRISPR-based technique [12]. Another example, the 10 kb isobutanol biosynthetic pathways were able to scarlessly insert into the genome with an efficiency of 50% [13]. However, the efficiencies and the fragments size of these integrations do not meet the requirement of some engineering goals.

**Fig. 1:**
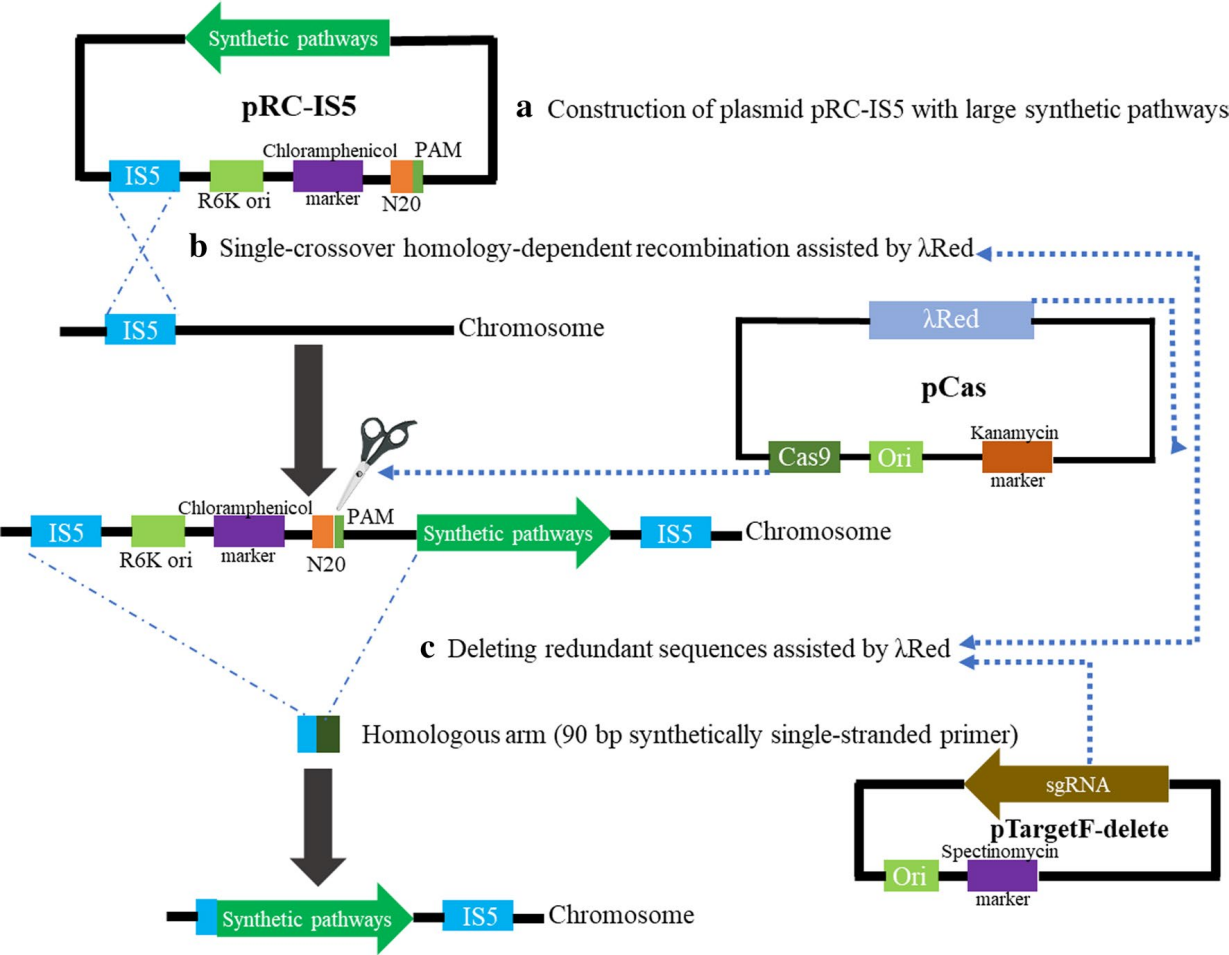
Outline of the λ-Red assisted homology-dependent recombination for large synthetic pathways integration in *Escherichia coli*. **a** Construction of plasmid pRC-IS5 with large synthetic pathways. pRC-IS5 (including R6K and a homologous region) replicates normally in *E. coli* with the expression of pir + protein and the plasmid replication was restricted in normal *E. coli*. **b** Single-crossover HDR assisted by λRed. The vector pRC-IS5 was introduced into the host which harbored pCas with the expression of Exo, Beta, and Gam, and then selection was conducted with the addition of chloramphenicol. Single crossover produced homology-dependent insertion events, where the entire vector pRC-IS5 was integrated into the chromosome at the target locus. A simple screening step by PCR diagnosis could identify the desired mutant. **c** Deleting redundant sequences assisted by λ-Red. The gRNA plasmid pTargetF-delete and the donor template were electroporated into the competent cells harbored plasmid pCas with the expression of Cas9 nuclease and λ-Red protein, and then the selection was carried out using kanamycin and spectinomycin. λ-Red mediated deletion at the lagging strand of the replication fork produced homologous recombination, where the redundant sequences were deleted

**Fig. 2:**
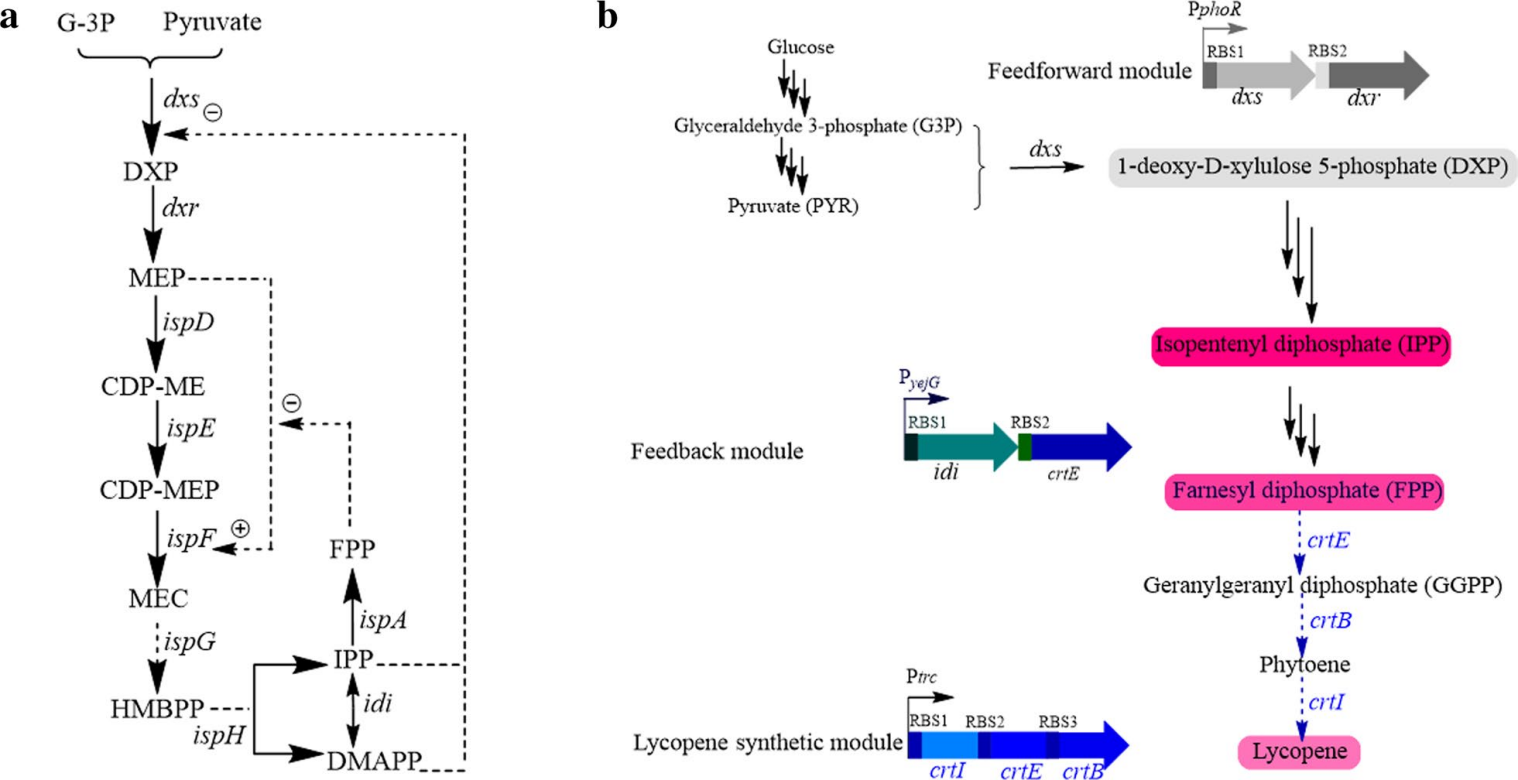
**a** MEP pathway and related metabolism showing the major metabolic regulatory points. d-glyceraldehyde 3-phosphate (G-3P);1-deoxy-d-xylulose 5-phosphate (DXP); methylerythritol 4-phosphate (MEP); diphosphocytidyl methylerythritol (CDP-ME); diphosphocytidyl methylerythritol 2-phosphate (CDP-MEP); hydroxymethylbutenyl diphosphate (HMBPP); isopentenyl diphosphate (IPP); dimethylallyl diphosphate (DMAPP); farnesyl diphosphate (FPP). **b** The limiting step for lycopene production was divided into three modules. The feedforward module including *dxs* and *dxr*, feedback module including *idi* and *crtE* and lycopene synthetic module including *crtI*, *crtE* and *crtB* for lycopene production

In this study, we developed a new strategy for integration of large synthetic pathways into *E. coli* W3110 genome (Fig. 1). To achieve this, we harnessed λ-Red and CRISPR/Cas9 system to increase recA-mediated HDR efficiency and delete redundant sequences, respectively. To demonstrate the feasible with which this system could be applied to genetical applications, we integrated the optimized synthetic pathways which combined limiting step in MEP pathway and lycopene synthetic pathway into *E. coli* W3110 genome using our system (Fig. 2). This approach enabled integration of synthetic pathways as large as 12 kb with efficiency of 100%. Consequently, we were able to obtain a strain capable of producing lycopene in a single step and the yields were increased 106 and 4.4-fold compared to the initial strain and the reference strain, respectively. Concerning the integrated DNA fragment size and the integrated efficiency, we ensured that our platform will be useful for metabolic engineering and synthetic biology in *E. coli*.

## Results

### Design of the λ-Red-assisted homology-dependent recombination for large synthetic pathway integration

The workflow of present strategy was illustrated in Fig. 1. The genome editing procedure was performed via plasmid pRC-IS5 and pCas. The plasmid pRC-IS5 contained the fragmentary IS5 sequence, the narrow-host-range replicon R6K, the chloramphenicol marker, the large synthetic pathways and a gRNA recognition region (N20PAM). The N20PAM sequence from *Saccharomyces cerevisiae* was used for reducing the off-target frequency. The plasmid pCas containing the λ-Red and CRISPR/Cas9 system [14]. When conducted the editing processes, the vector pRC-IS5 was inserted into the *E. coli* W3110 genome by recA-mediated HDR assisted by λ-Red. Then a Double-stranded break (DBS) which induced λ-Red promoted HDR by donor template (90 bp synthetically single-stranded primer) was created by Cas9 nuclease to accomplish the editing processes (Fig. 1). Finally, the redundant sequences including *IS5* sequence, chloramphenicol marker and R6K were deleted.

To facilitate this platform for genome editing, λ-Red recombinases (Exo, Beta, and Gam) were expressed to facilitate the recA-mediated HDR. The plasmid pCas (MolecularCloud Cat. No.: MC_0000011) was used to fulfill this function, in which λ-Red was induced via the inducible promoters *pBAD* and the CRISPR/Cas9 systems was controlled by the native promoter. The λ-Red system was induced by 0.2% l-arabinose for the chromosomal insertion of the pRC-IS5, while the CRISPR/Cas9 system was expressed for the generation of a DSB at the universal N20PAM to remove the redundant sequences.

### Construction of a model synthetic pathway for integration

The biosynthesis of lycopene was extensively studied and the synthetic pathway for lycopene was usually used as the model pathway in metabolic engineering and synthetic biology [15, 16]. Thus, the production of lycopene was chosen as a model pathway in this work. Many studies have demonstrated that the first two and last two steps of the MEP pathway were the limiting steps for lycopene production [17–20]. Based on the metabolic regulation of MEP pathway [21] (Fig. 2a), we divided the model synthetic pathways into three modules which comprised feedforward module including *dxs* (Gene ID: 938609) and *dxr* (Gene ID: 939636), feedback module including *idi* (Gene ID: 938985) and *crtE* and lycopene synthetic module including *crtI*, *crtE* and *crtB* (GenBank: CP002191) (Fig. 2b). Many previous studies have proved that the carbon and energy flux were directed to cell growth in the early growth phase and later redirected to synthetic pathways to support target product formation in growth regulated pathways [22, 23]. The transcriptome analysis along with the growth phase has been done by a previous work [24]. In order to construct growth regulated pathways, we have picked the promoters which maintained low expression at exponential phase and strong upregulated when cultured to the end of exponential phase and held high expression latterly for these three modules based on the transcriptome data along with the growth phase (GSE102672). We defined each promoter as the 600 bp upstream of the ribosome-binding site (RBS) of its corresponding coding sequence, since these regions generally contained most regulatory sequences [25]. To avoid the influence by RBS site, the same Shine–Dalgarno sequence was used for each module.

**Fig. 3:**
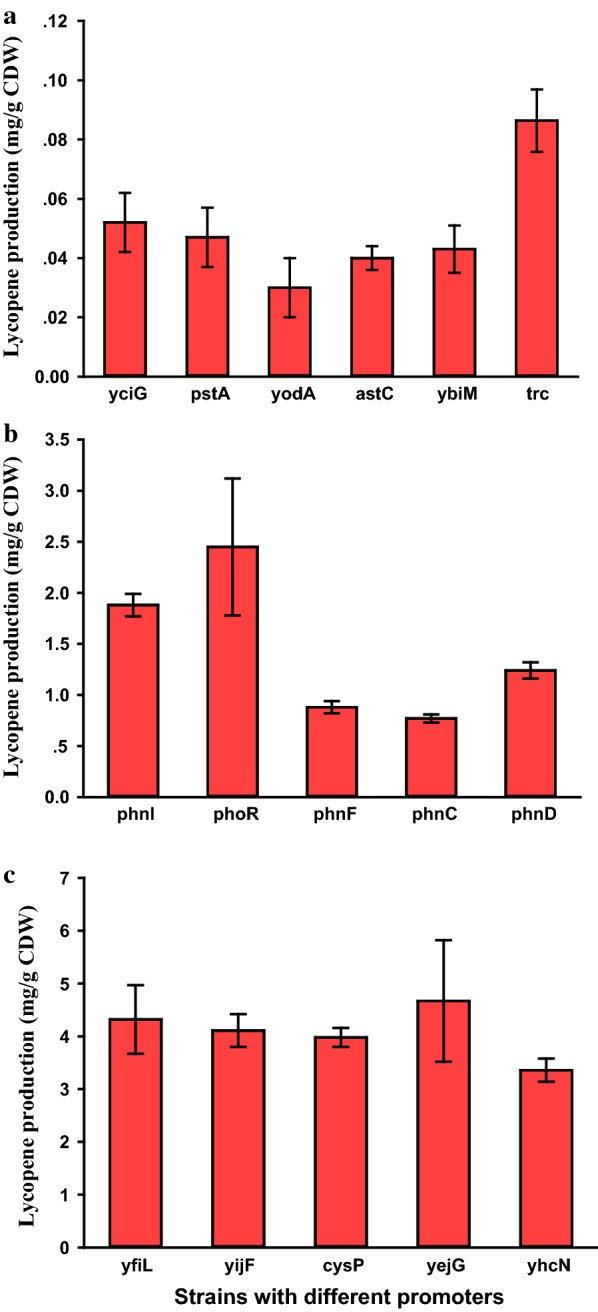
Promoters characterization of three modules for lycopene biosynthesis. **a** Selection promoters for lycopene synthetic modules (including *crtI*, *crtE* and *crtB*). Lycopene synthetic modules were overexpressed in *E. coli* W3110 with the native MEP pathway. trc, EC101; yciG, EC102; pstA, EC103; yodA, EC104; astC, EC105; ybiM, EC106. **b** Selection promoters for feedforward modules (including *dxs* and *dxr*). Feedforward modules were overexpressed along with lycopene synthetic modules. phnI, EC201; phoR, EC202; phnF, EC203; phnC, EC204; phnD, EC205. **c** Selection promoters for feedback modules (including *idi* and *crtE*). Feedback modules were overexpressed along with lycopene synthetic modules. yfiL, EC301; yijF, EC302; cysP, EC303; yeiG, EC304; yhcN, EC305. Each value represents the average ± SD of three biological replicates

As shown in Fig. 3, the *trc* (from pTrc99a [26]) was the best promoter for lycopene synthetic module, indicating that stronger promoter was needed for lycopene production. Similar result from previous work suggested that efficient lycopene production relied on maintaining high levels of lycopene synthase [27]. Based on the shake flask fermentations, the *PphoR* and *PyejG* were the best promoters for feedforward and feedback modules, respectively. Thus, promoters *trc*, *PphoR* and *PyejG* were chosen for the construction of the model synthetic pathways.

### Integration of a 12 kb DNA module into *E. coli* W3110 genome

**Fig. 4:**
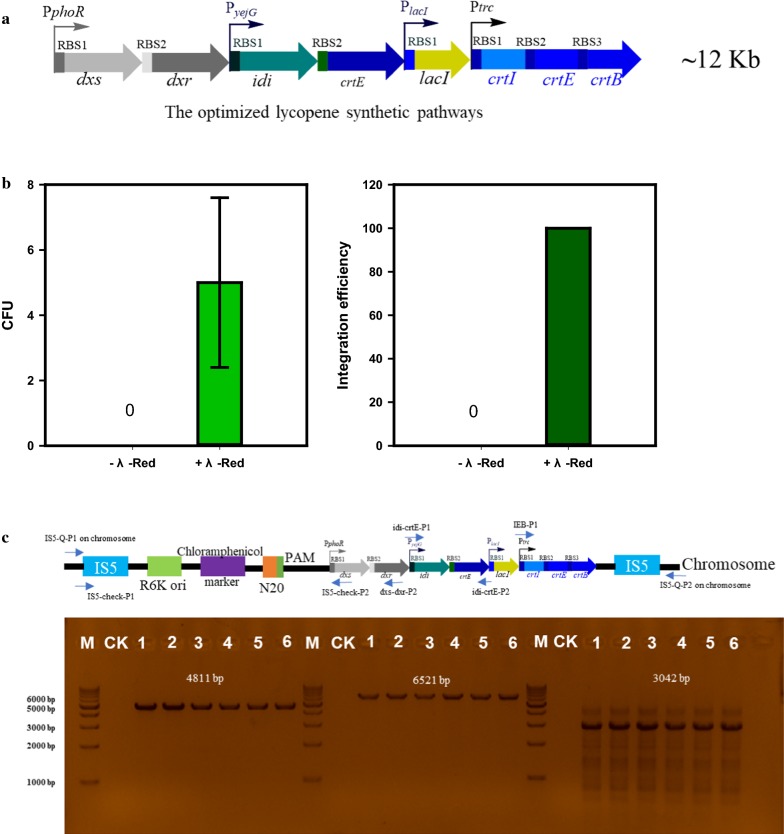
Integration of a 12 kb DNA module into *E. coli* W3110 genome. **a** The optimized lycopene synthetic pathways in pRC-IS5. **b** Colony forming unit (CFU, indicated the number of colonies on the selective plates with 34 μg mL^−1^ chloramphenicol after one experiment of integrating optimized lycopene synthetic pathways into *E. coli* W3110) and integration efficiency with or without adding arabinose to induce λ-Red. **c** PCR confirmation of the integration of the optimized lycopene synthetic pathways using primers IS5-Q-P1 and dxs-dxr-P2 for feedforward module (6521 bp), IEB-P1 and IS5-Q-P2 for lycopene synthetic module (4811 bp), idi-crtE-P1 and idi-crtE-P2 for feedback module (3042 bp). M: DNA marker; CK: *E. coli* W3110; 1, 2, 3, 4, 5, 6: colonies from the plates after chromosomal integration

**Fig. 5:**
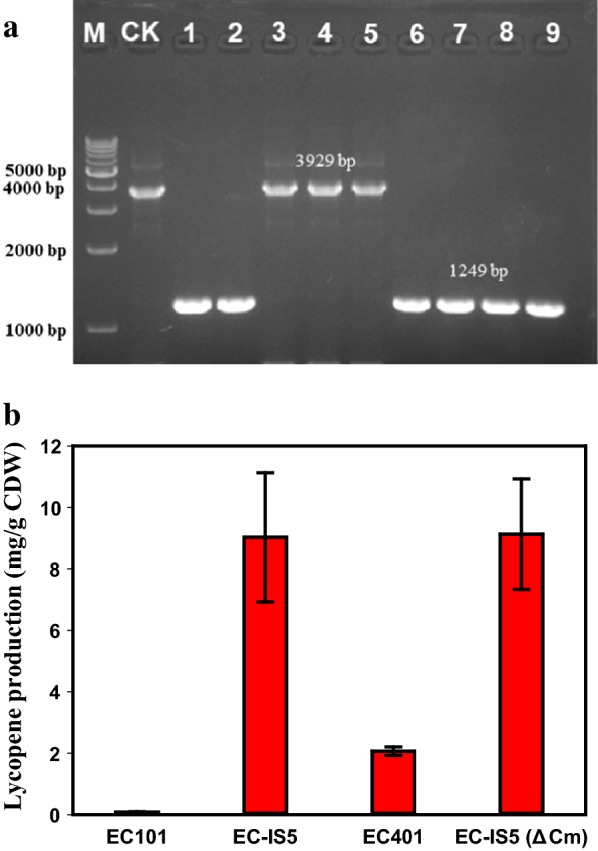
Deletion of redundant sequences with CRISPR-Cas9 system. **a** PCR confirmation of the deletion of redundant sequences using primers IS5-check-P1 and IS5-check-P2. The decrescent bands indicated the successful deletion of redundant sequences. M: DNA marker; CK: strain without deletion of redundant sequences; 1,2,3,4,5,6,7,8,9: colonies from the plate after editing using CRISPR-Cas9 system. **b** The lycopene yields of shake flask fermentation of strain EC101, EC-IS5, EC401 and EC-IS5 (ΔCm). Each value represents the average ± SD of three biological replicates

To verify the efficacy of the designed platform for integration of large fragments, we used the above synthetic pathways (~ 12 kb) as a model module to integrate it into *E. coli* W3110 genome. We divided the plasmid pRC-IS5 into four segments including three modules and the vector backbone. Firstly, we obtained the integrative vector pRC-IS5 through Gibson assembly method (Additional file 1: Fig. S1) [28]. Subsequently, pRC-IS5 was integrated into the *IS5* locus through recA-mediated HDR assisted by λ-Red. Consequently, the optimized lycopene synthetic pathways (~ 12 kb) was integrated into *E. coli* W3110 genome. The correct integration was verified by the red color and colony PCR, and the edited strain was designated as EC-IS5. We found that all the colonies on the plates were red colored with λ-Red and there was no colony without adding arabinose to induce λ-Red (Additional file 1: Fig. S2). Then the red colonies were further verified by colony PCR (Fig. 4c). This result indicated that the λ-Red system was crucial for recA-mediated HDR when generated large pathway integration. Strain EC-IS5 produced 9 mg/g CDW of lycopene in the shake flask fermentation, whereas the plasmid-based strain EC101 and EC401 produced 0.086 and 2.1 mg/g CDW of lycopene, respectively (Fig. 5). EC-IS5 produced 105-fold increase of lycopene yield through integrating the optimized lycopene synthetic pathways into genome compared with the initial strain (EC101). These results indicated that this strategy which combined modular pathway engineering and integrated strategy represented a remarkable synergy.

### Deletion of redundant sequences with CRISPR-Cas9 system

We next cultivated EC-IS5 in medium supplemented with kanamycin at 30 °C to maintain the pCas plasmid and made the competent cell washed by 10% glycerol. To obtain marker-free strains, the competent EC-IS5 was transformed with 90 bp synthetically single-stranded donor template and plasmid pTargetF-delete (constructed based on pTargetF-cadA [14]) using electroporation and then were spread on the LB plate with kanamycin and spectinomycin. The deletion efficiency of the redundant sequences was exceeded 70% after an overnight incubation (Additional file 1: Fig. S3). This feature might facilitate iterative genome editing. Then the final strain EC-IS5 (ΔCm) was used for lycopene production without antibiotic maintenance. As shown in Fig. 5, strain EC-IS5 (ΔCm) produced 9.1 mg/g CDW lycopene, which was 4.4-fold of the reference strain (EC401). This result confirmed that chromosomal integration shown great advantage than plasmid-based method.

## Discussion

In a previous study, we developed a platform for chromosomal integration (~ 1.5 kb) in *E. coli* for xylitol production using recA-mediated HDR [29]. In preliminary experiment before this study, we attempted to integrate a larger pathway (~ 7 kb, only including *crtI*, *crtB* and *crtE*) into *E. coli* chromosome using recA-mediated HDR. Nevertheless, we could not get any colonies using the above method and CRISPR/Cas9 system [14]. Alonso-Gutierrez and colleagues attempted to integrate a synthetic pathway comprised terpene synthase (~ 12 kb) into *E. coli* DH1 genome using assistant plasmid which could mediate chromosomal integration by λ-Red promoted HDR [7]. However, they could not get the expected integration through this system. They claimed that the large size of the synthetic pathway and the complicacy of the pathway might be the most probable explanation for these failing attempts. As an alternative, they divided the synthetic pathway into three segments (shorter than 5 kb) and integrated them through three rounds of integration to achieve the final integration [30].

*Escherichia coli* was highly dependent on a homologous recombination to repair DBS in the chromosome. λ-Red promoted HDR successfully supplemented the low efficiency of the *E. coli* native repair system and, thus, succeeded in genome editing, while single DSB could not be repaired without λ-Red [14]. The recA-mediated HDR is another form of allelic exchange [31]. However, this recombination is insufficient for large pathway integration (Fig. 4b). Fortunately, we successfully rescued the low efficiency of recA-mediated HDR by using λ-Red. RecA is one of DNA strand exchange proteins which are essential for homologous recombination. In vivo, RecA preferentially binds to ssDNA (double stranded DNA (dsDNA) breaks or ssDNA gaps in replication forks stall), and then the assembly of a presynaptic filament of RecA on the ssDNA was generated during homologous recombination, which in turn uses the ssDNA sequence to search for a homologous region in the dsDNA [32]. Meanwhile, the λ-Red system consists of several genetic components (Exo, Beta, and Gam) and Beta also binds to the ssDNA [2]. Although we do not know what is the real mechanism, we speculated that Beta would likely facilitate rescuing the low activity of recA-mediated HDR for large synthetic pathway.

Recent years, CRISPR/Cas9 based genome editing has been obtained great progresses in *E. coli*. However, chromosomal integration of large DNA modules was still limited by low efficiency and integration size compared with chromosomal integration of DNA modules shorter than 2000 bp. To alleviate these issues, Li and colleagues developed a platform with multiple step integration of divided segments [33]. They found that the integration efficiency decreased gradually with the increasing size of the modules, and the efficiency for a 7 kb DNA module was about 10% which was similar to that in *E. coli* MG1655 [34]. To facilitate the high efficiency and the preparation of the DNA modules, about 3–4 kb of the entire DNA module was optimal. They could insert a 15.4 kb synthetic pathway which contained several crucial genes for uridine biosynthesis into *E. coli* W3110 genome with five steps of integration [33]. However, this strategy needed many rounds of integration to access the final goals.

Using our platform, we were able to obtain a strain capable of producing lycopene in a single step and the production yields were increased 106 and 4.4-fold compared to the initial strain (EC101) and the reference strain (EC401), respectively (Additional file 1: Fig. S4). This study clearly demonstrate that our platform was quite feasible and useful for constructing microbial cell factories which needed large synthetic pathways. Therefore, we concluded that the recA-mediated HDR integration aided by λ-Red in this study was relatively practical for metabolic engineering (Table 1). Furthermore, we speculated that our platform facilitated integration of large synthetic pathways could be applied to other prokaryotic microorganism to achieve stable strains for chemical production, for that insertion sequences were widely distributed in many microorganism [35].

**Table 1:** Methods for the integration of large DNA module into *E. coli* chromosome

Method	Technique feature	Integration efficiency (%)	Integration sites	Maximum integration size (kb)	Rounds of integration	Marker left or markerless	Reference
I-SceI endonuclease	Combined λ-Red and I-SceI Cleavage	19.2–100	Desired locus	7	One	Markerless	[5]
λ-Red recombination	λ-Red assisted	0–50	Desired locus	7.3	One	Markerless	[7]
λ-Red recombination	Combined λ-Red and CRISPR/Cas9	10	Desired locus	7	One	Markerless	[33] [9]
		60		15.4	Five	Markerless	
λ-Red recombination	λ-Red assisted	Not provide	*fliK*	15	Four	Markerless	
		Not provide	*fliK*	50	Seven	Markerless	
CRISPR/Cas9	Combined λ-Red and CRISPR/Cas9	60	Desired locus	7	One	Markerless	[12]
CRISPR/Cas9	Combined λ-Red and CRISPR/Cas9	50	Desired locus	10	One	Markerless	[13]
RecA homologous recombination	Combined λ-Red, RecA and CRISPR/Cas9	100	*IS5*	12	One	Markerless	This study

## Conclusions

We have developed a useful platform for integration of large synthetic pathways into *E. coli* W3110 genome. Taking advantage of the λ-Red promoted HDR and the Cas9 nuclease, only the integrative vector is needed to construct for each round of integration. Another characteristic is that stable strains can be obtained by integration of large synthetic pathways that are responsible for valuable chemicals biosynthesis. In order to verify the feasibility of our platform, a 12 kb DNA module contained several key genes for lycopene biosynthesis was integrated into the *E. coli* W3110 chromosome. The production yields were increased 106 and 4.4-fold compared to the initial strain (EC101) and the reference strain (EC401), respectively. Our platform has been proven to be practical in *E. coli* and would be adapted for the production of valuable chemicals.

## Methods

### Strains and culture medium

Strains and plasmids used in this study are listed in Table 2. *E. coli* strain DH5α and DH5α λpir (*pir*^+^ for propagating R6K ori) were used for the construction of the plasmids. *E. coli* W3110 was used for chromosomal integration. Strains were cultured in Luria–Bertani (LB) medium supplemented with 100 μg mL^−1^ ampicillin, 50 μg mL^−1^ kanamycin, 50 μg mL^−1^ spectinomycin or 34 μg mL^−1^ chloramphenicol when needed at 37 °C or 30 °C with shaking at 200 rpm.

**Table 2:** *Escherichia coli* strains and plasmids used in this study

Strain/plasmid	Description	Source
*Strains*		
DH5ɑ	*supE44 ΔlacU169 (φ80 lacZΔM15) hsdR17 recA1 endA1 gyrA96 thi*-*1 relA1*	Invitrogen
DH5α λpir	*supE44 ΔlacU169 (φ80 lacZΔM15) hsdR17 LAMpir U169 recA1 endA1 gyrA96 thi*-*1 relA1*	Lab stock
W3110	Wide type, *λ*-*F*-*mcrA mcrB IN (rrnD*-*rrnE)1*	DSM5911
EC101	W3110 with plasmid pET-trc-IEB	This study
EC102	W3110 with plasmid pCDF-yciG-IEB	This study
EC103	W3110 with plasmid pCDF-pstA-IEB	This study
EC104	W3110 with plasmid pCDF-yodA-IEB	This study
EC105	W3110 with plasmid pCDF-astC-IEB	This study
EC106	W3110 with plasmid pCDF-ybiM-IEB	This study
EC201	W3110 with plasmid pACYC-phnI	This study
EC202	W3110 with plasmid pACYC-phoR	This study
EC203	W3110 with plasmid pACYC-phnF	This study
EC204	W3110 with plasmid pACYC-phnC	This study
EC205	W3110 with plasmid pACYC-phnD	This study
EC301	W3110 with plasmid pACYC-yfiL	This study
EC302	W3110 with plasmid pACYC-yijF	This study
EC303	W3110 with plasmid pACYC-cysP	This study
EC304	W3110 with plasmid pACYC-yejG	This study
EC305	W3110 with plasmid pACYC-yhcN	This study
EC401	DH5α λpir with plasmid pRC-IS5	This study
EC-IS5	W3110 with the integration of plasmid pRC-IS5	This study
EC-IS5(ΔCm)	EC-IS5 with the deletion of *Cm*	This study
*Plasmids*^a^		
pCDFDuet-1	pCloDF13-derived vector; T7 promoter, Str^R^	Lab stock
pET-30a-trc	pBR322-derived vector; trc promoter, Kmr^R^	Lab stock
pACYCDuet-1	p15A-derived vector; T7 promoter, Cmr^R^	Lab stock
pTrc99a	pBR322-derived vector; trc promoter, Amp^R^	[26]
pRC43	Including R6K ori, *Cm*, *IS5* sequence	[29]
pCas	*repA101(Ts) kan Pcas*-*cas9 ParaB*-*Red lacIq Ptrc*-*sgRNA*-*pMB1*	[14]
pTargetF-cadA	pMB1 aadA sgRNA-cadA	[14]
pET-trc-IEB	Lycopene synthetic module under the trc promoter	This study
pCDF-yciG-IEB	Lycopene synthetic module under the yciG promoter	This study
pCDF-pstA-IEB	Lycopene synthetic module under the pstA promoter	This study
pCDF-yodA-IEB	Lycopene synthetic module under the yodA promoter	This study
pCDF-astC-IEB	Lycopene synthetic module under the astC promoter	This study
pCDF-ybiM-IEB	Lycopene synthetic module under the ybiM promoter	This study
pACYC-phnI	Lycopene synthetic module under the yciG promoter, feedforward module under the phnI promoter	This study
pACYC-phoR	Lycopene synthetic module under the yciG promoter, feedforward module under the phoR promoter	This study
pACYC-phnF	Lycopene synthetic module under the yciG promoter, feedforward module under the phnF promoter	This study
pACYC-phnC	Lycopene synthetic module under the yciG promoter, feedforward module under the phnC promoter	This study
pACYC-phnD	Lycopene synthetic module under the yciG promoter, feedforward module under the phnD promoter	This study
pACYC-yfiL	Lycopene synthetic module under the yciG promoter, feedback module under the yfiL promoter	This study
pACYC-yijF	Lycopene synthetic module under the yciG promoter, feedback module under the yijF promoter	This study
pACYC-cysP	Lycopene synthetic module under the yciG promoter, feedback module under the cysP promoter	This study
pACYC-yejG	Lycopene synthetic module under the yciG promoter, feedback module under the yejG promoter	This study
pACYC-yhcN	Lycopene synthetic module under the yciG promoter, feedback module under the yhcN promoter	This study
pTargetF-delete	gRNA for N20PAM	This study
pRC-IS5	Including R6K ori, *Cm*, fragmentary *IS5* sequence, Lycopene synthetic module under the trc promoter, feedforward module under the phoR promoter and feedback module under the yejG promoter	This study

^a^ Amp^R^: ampicillin; Kan^R^: kanamycin; Str^R^: Streptomycin; Cm: chloramphenicol; R:resistance

### Plasmid construction

Primers for construction of various plasmids are listed in Additional file 1: Table S1. Plasmids for expression of heterologous lycopene synthesis pathway are based on pCDFDuet-1 or pET-30a-trc, plasmids for expression of feedforward module and feedback module were based on pACYCDuet-1. Plasmid containing the large synthetic pathways is divided into several small fragments, including three modules and the vector backbone. All the plasmids were constructed according to the protocol of ClonExpress MultiS One Step Cloning Kit (Vazyme, China).

### Chromosomal integration procedure

Briefly, the host strain *E. coli* W3110 was transformed with pCas and then was prepared for competent cells with λ-Red recombinase induction by L-arabinose according to the protocol [36]. Immediately, 100 μL of the competent cells was mixed with 300 ng of plasmid pRC-IS5 in MicroPulser (Eppendorf). After electroporation (2.5 kV, 5 ms), the competent cells were suspended in 1 mL LB broth quickly. After 6 h incubated at 30 °C, cells were centrifuged and resuspended in 0.1 mL sterile water. Then the cells were spread on LB agar plates with chloramphenicol. After genome editing, the colonies on the plates were identified by red color and colony PCR with primers IS5-Q-P1, dxs-dxr-P2, idi-crtE-P1, idi-crtE-P2, IEB-P1 and IS5-Q-P2 which straddling the synthetic pathway on chromosome (Additional file 1: Table S1). The editing efficiency was calculated as the number of colonies with red color divided by the number of all the colonies. The correct strain was transferred into LB broth with kanamycin, and was prepared as electrocompetent cells with the expression of Cas9 nuclease and λ-Red proteins. Donor template (90 bp synthetically single-stranded primer) and plasmid pTarget-delete (including the gRNA sequence) were electroporated into the competent cells and then the cells were spread on the LB plate with kanamycin and spectinomycin. Deletion of the redundant sequences was identified by chloramphenicol sensitive and colony PCR using primers IS5-check-P1 and IS5-check-P2 (Additional file 1: Table S1).

### Shake flask cultures and analysis of lycopene

For shake flask fermentation, a single colony selected from a fresh LB agar plate was grown overnight in 5 mL of LB broth in a shaker at 37 °C for overnight growth, then 1 mL of the preculture was inoculated into 250 mL shake flask containing 50 mL 2 × TY medium with 4% glycerol and grown at 30 °C for 48 h. Individual flasks were stopped at regular times to determine biomass and lycopene yields. Extraction of carotenoid was as described by literature with some modifications [37]. Briefly, cells were harvested by centrifugation at 8000*g* for 5 min, and then were suspended in 1 mL of acetone. The lysate was incubated at 55 °C for 15 min and centrifuged at 12,000*g* for 20 min. The acetone supernatant was transferred into a clean tube for measuring lycopene. The lycopene content of the extracts was determined by UV/Vis spectrometer (PerkinElmer Lambda 45) at 470 nm. Spectra was recorded in acetone using an A 1% 1 cm of 3450 [38]. The yields of lycopene were expressed as mg per g cell dry weight (mg/g CDW).

## Supplementary information

**Additional file 1.** Additional tables and figures.

## Data Availability

The datasets and materials used during the current study are available from the corresponding author on reasonable request.
