# Supplementary material for: Homology-dependent recombination of large synthetic pathways into E. coli genome via λ-Red and CRISPR/Cas9 dependent selection methodology
Source: Microb Cell Fact. 2020 May 24;19:108. doi: 10.1186/s12934-020-01360-x (PMC7245811; doi:10.1186/s12934-020-01360-x)

**Supplementary Materials**

**Homology-dependent recombination of large synthetic pathways into *E. coli* genome via λ-Red and CRISPR/Cas9 dependent selection methodology**

Buli Su, Dandan Song, Honghui Zhu*

State Key Laboratory of Applied Microbiology Southern China, Guangdong Provincial Key Laboratory of Microbial Culture Collection and Application, Guangdong Microbial Culture Collection Center (GDMCC), Guangdong Institute of Microbiology, Guangdong Academy of Sciences, Guangzhou 510070, People’s Republic of China.

*Corresponding author

Honghui Zhu: E-mail: zhuhh@gdim.cn

Tel: +86-020-87137669, Fax: +86-020-87685699

**Table S1.** Primers used in this study. Homologous overhang-nucleotides were underlined, RBS were bold and italics.

| Primers | Sequence (5’-3’) |  |
| --- | --- | --- |
| trc-30a-P1  trc-30a-P2  crtI-P1  crtI-P2  crtE-P1  crtE-P2  crtB-P1  crtB-P2  CDF-P1  CDF-P2  I-E-B-P1  I-E-B-P2  yodA-P1  yodA-P2  astC-P1  astC-P2  pstA-P1  pstA-P2  yciG-P1  yciG-P2  ybiM-P1  ybiM-P2  pACYC-P1  pACYC-P2  yciG-IEB-P1  yciG-IEB-P2 | Primers for construction of pET-trc-IEB  ttctctgcttaaataactagcataaccccttgg  agaaacggtcatatgtatatctccttcttaaagttaa  ggagatatacatatgaccgtttcttctgcttctccgc  ttgttttcctccttgttccgtattttagtcgtcagcagcagcacccagc  ***aatacggaacaaggaggaaaacaa***atgcgtccggaactgctggaacgtg  ggtatatctccttcttaaagttaattaagcttcacgggtagccagttca  ***ttaactttaagaaggagatatacc***atgaccgactgcgctccgccggctc  tatgctagttatttaagcagagaaagaacgcagttcc  Primers for constructing the plasmid of IEB module  aataactagcataaccccttggg  agcggttcagtagaaaagatc  aacttttaagaaggagatataccatgaccgtttcttctgcttctccgc  cccaaggggttatgctagttattttaagcagagaaagaacgcagttcc  gatcttttctactgaaccgcttggagctgggcgtgaataatc  ggtatatctccttcttaaaagtttgttatattgtaacatatagcaaaatgc  gatcttttctactgaaccgcttagccgagcttcgccacctcttc  ggtatatctccttcttaaaagttgttaataataagtaatgtttgcgctgta  gatcttttctactgaaccgctatctttgcgccgctgttcgcc  ggtatatctccttcttaaaagtttcataaacttcgatgcggcgagg  gatcttttctactgaaccgctggttgcgaagctgaaagagtac  ggtatatctccttcttaaaagtttcattaatatgagtgttgtgtgtcg  gatcttttctactgaaccgctccagaatgctggcattcgc  ggtatatctccttcttaaaagtttagtgcctgataagtgtaggtcg  Primers for constructing the plasmid of dxs-dxr module  aataactagcataaccccttggg  actctttcagcttcgcaaccacttcaggtgctacatttgaagag  ggttgcgaagctgaaagagtac  caaaaaacccctcaagacccgt |  |
| dxs-P1  dxs-P2  dxr-P1  dxr-P2 | ***gtttaactttaagaaggagatatacc***atggatcttttatcaatacaggaccc  ttgttttcctccttattaaatcatgatccaattcctttgtgtgt  ***tttaataaggaggaaaacaa***atgaaaaatatttgtcttttaggagcaacaggatca  cccaaggggttatgctagttattttatgtgagtattgaattgacgtatcc |  |
| phnC-P1 | gggtcttgaggggttttttgactgaggtcaccatcatgccg |  |
| phnC-P2 | ggtatatctccttcttaaagttaaaccatcgtggcggattattgtga |  |
| phnD-P1 | gggtcttgaggggttttttgcagcgcgaaggccgcctg |  |
| phnD-P2 | ggtatatctccttcttaaagttaaacgcagctttcgcgttctct |  |
| phnF-P1 | gggtcttgaggggttttttgccgtcacgctgcaaatcgccg |  |
| phnF-P2 | ggtatatctccttcttaaagttaaacgtgcttacgcaaccgttgag |  |
| phnI-P1 | gggtcttgaggggttttttgcgcggagacaacgcatgac |  |
| phnI-P2 | ggtatatctccttcttaaagttaaaccggaatagccagcaggcgc |  |
| phoR-P1 | gggtcttgaggggttttttgcctcgactggatgttacctggc |  |
| phoR-P2  idi-P1  idi-P2  crtE-P1  crtE-P2  cysP-P1  cysP-P2  yijF-P1 | ggtatatctccttcttaaagttaaacgccctgctctgcgtccgatgag  Primers for constructing the plasmid of idi-crtE module  ***aaatacggaacaaggaggaaaacaa***atgactcgagcagaacgaaaaag  gtatatctccttcttttatcgcacactatagcttgatgtattg  tatagtgtgcgataa***aagaaggagatatacat***atgcgtccggaactgctggaacg  cccaaggggttatgctagttattttaagcttcacgggtagccagttca  gggtcttgaggggttttttgacaaaatcgctggcggtgga  ttgttttcctccttgttccgtatttagccatcatatttataacggacgtg  gggtcttgaggggttttttgtgaccgctatcccggacgc |  |
| yijF-P2  yejG-P1  yejG-P2  yfiL-P1  yfiL-P2  yhcN-P1  yhcN-P2  phoR-dxs-dxr-P1-3  phoR-dxs-dxr-P1-2  phoR-dxs-dxr-P1  phoR-dxs-dxr-P2  yejG-idi-crtE-P1  yejG-idi-crtE-P2  IEB-P1  IEB-P2  IS5-R6K-CM-P1  IS5-R6K-CM-P2  IS5-R6K-CM-P2-2  IS5-Q-P1  dxs-dxr-P2  idi-crtE-P1  idi-crtE-P2  IEB-P1  IS5-Q-P2  IS5-check-P1  IS5-check-P2  IS5-R6K-Cm-delete  delete-CRISPR-P1  delete-CRISPR-P2 | ttgttttcctccttgttccgtatttcacagattcgaccttccgggc  gggtcttgaggggttttttggctggtggttggcgttgcgg  ttgttttcctccttgttccgtatttcatcaattttaacacatcatcaacaatccgtc  gggtcttgaggggttttttggcaatttacgcgcgatctgca  ttgttttcctccttgttccgtatttgcggattgttttcaaagggagtgt  gggtcttgaggggttttttgccagtccattgaagaatctggtgc  ttgttttcctccttgttccgtatttggcctttgtttcgtgacccat  Primers for amplification of the phoR-dxs-dxr module  aaaatctcaaaaatctcaaaaggaaggtaactcttcaaatgtagcacctgaagt  ctcttcaaatgtagcacctgaagtgagctcagctttattgagtgg  gagctcagctttattgagtggcctcgactggatgttacctggc  tcagcaaaaaacccctcaagacc  Primers for amplification of the yeiG-idi-crtE module  ggtcttgaggggttttttgctgagctggtggttggcgttgcgg  caccaaacgtttcggcgagaagcttgactaccggaagcagtgtg  Primers for amplification of the lacI-trc-IEB-terminator module  gcttctcgccgaaacgtttggtgg  catgcgggacaagaaaatctcttatccggatatagttcctcctttcag  Primers for amplification of the IS5-R6K-Cm module  aagagattttcttgtcccgcatg  ccatgtccttagttcggcttctgcaaataaaacgaaaggctcagtcg  ttaccttccttttgagatttttgagattttccatgtccttagttcggcttctg  atgtttgtcatctggagccatagaacagg  agtattgaattgacgtatccccgggt  tggtgtcatccaatgcgatggcggt  cgtcactggtcccgccaccaaacgt  gacaattaatcatccggctcgtataa  tcatgtttgagccgattttttctcccg  aagagattttcttgtcccgcatggagcag  ccgagcatagagtgaatggcac  Deletion of redundant sequences  aagagattttcttgtcccgcatggagcagattctgccatggcaaactcttcaaatgtagcacctgaagtgagctcagctttattgagtgg  Primers for constructing the plasmid of pTargetF-delete  cagaagccgaactaaggacagttttagagctagaaatagc  tgtccttagttcggcttctgactagtattatacctaggac |  |

**Fig. S1.** The map of plasmid pRC-IS5.


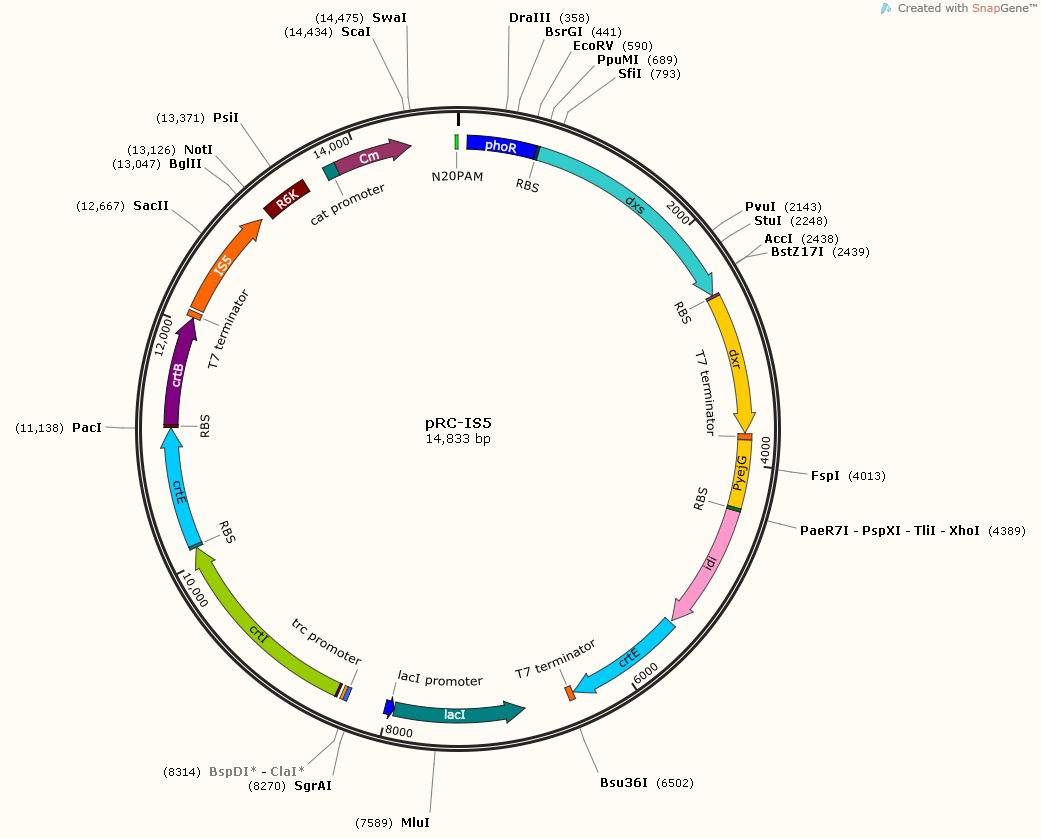


**Fig. S2.** The plate streaking cultivation after chromosomal integration procedure. The plate was cultivated on LB medium for two Days.


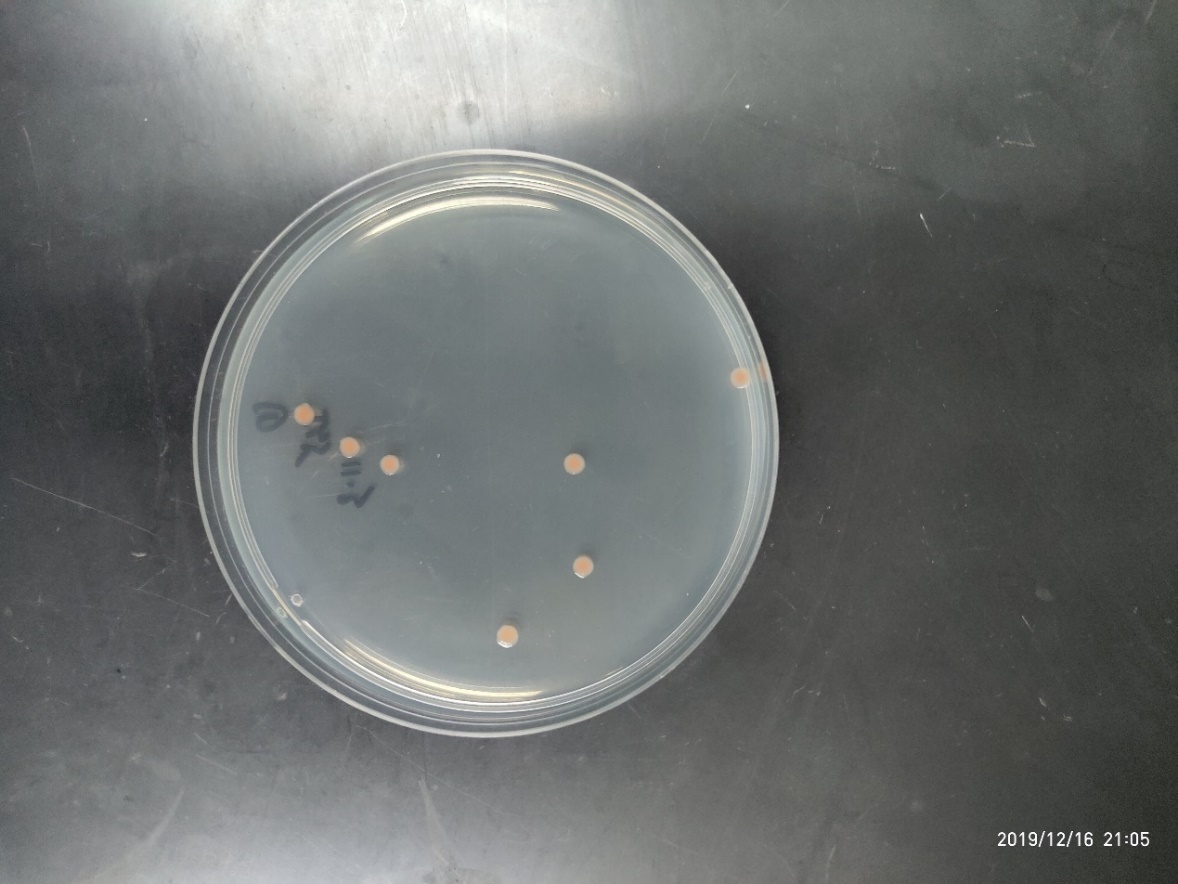


**Fig.** **S3.** Chloramphenicol sensitive of the edited strains. The plate was cultivated on LB medium for two Days.


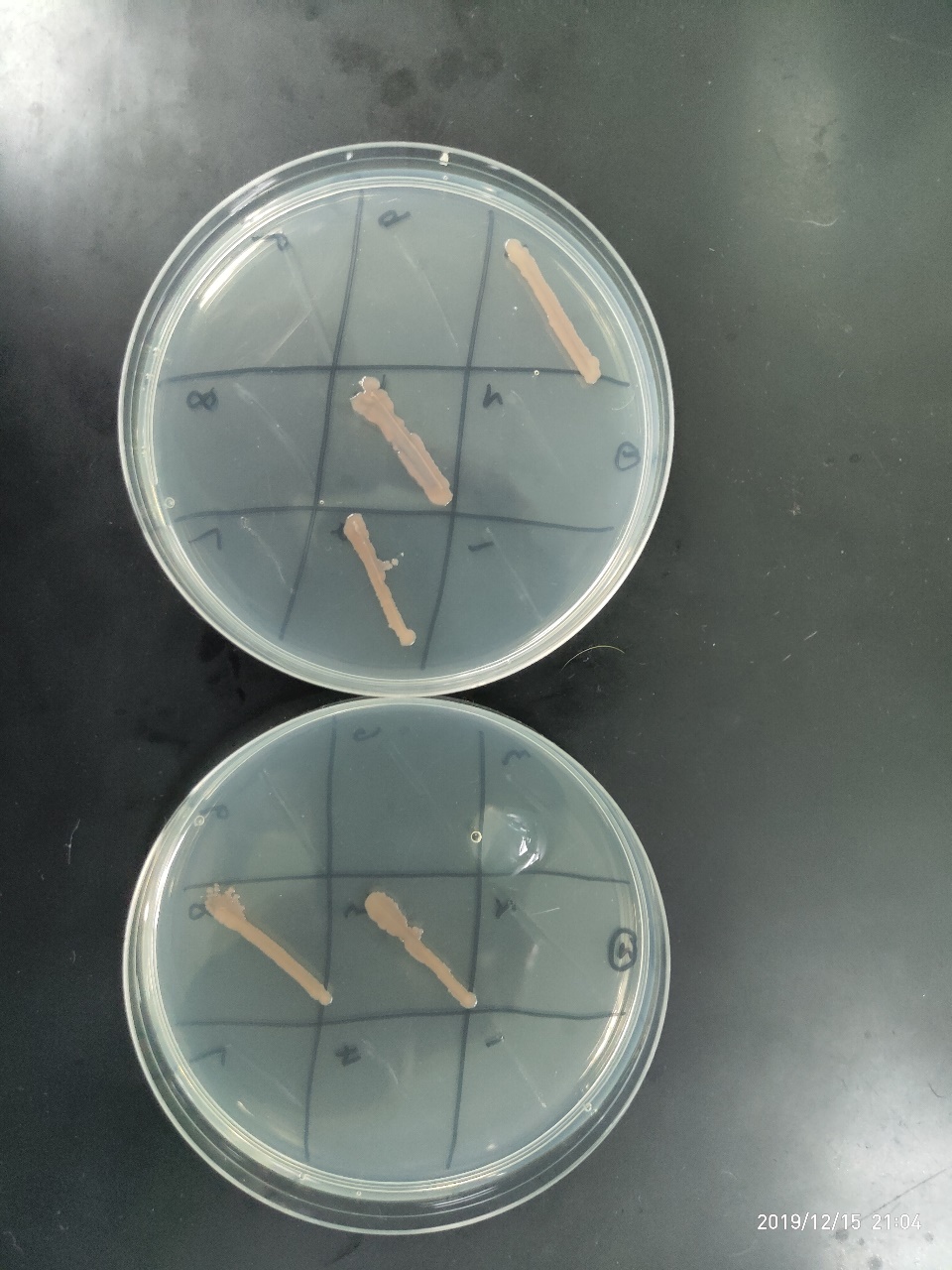


**Fig. S4.** Strains EC101, EC401 and EC-IS5 (ΔCm) after shake flask cultures.


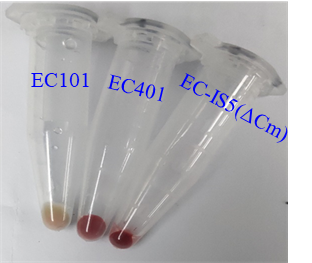

Supplement: Supplementary file 1 — Additional file 1. Additional tables and figures. [file 12934_2020_1360_MOESM1_ESM.docx]
